# Case Series: Natural History and Treatment of Thoracic Aortic Sarcoma

**DOI:** 10.1002/jso.28150

**Published:** 2025-05-15

**Authors:** Calvin L. Chao, Nicola M. Habash, Mark K. Eskandari

**Affiliations:** ^1^ Department of Surgery, Division of Vascular Surgery Northwestern University Feinberg School of Medicine Chicago Illinois USA

## Abstract

Aortic sarcoma is a rare and aggressive malignancy with a poor prognosis despite surgical resection and vascular reconstruction. We present a case series of patients with thoracic aortic sarcoma, highlighting three distinct clinical scenarios: primary resection, resection following prior thoracic aortic endograft, and nonoperative management. Our findings underscore key aspects of aortic sarcoma management, including its embolic potential, diagnostic challenges, and surgical considerations, particularly when endograft explant is required. Despite limited survival, we advocate for complete resection and vascular reconstruction with adjunctive chemoradiotherapy when feasible. Multidisciplinary collaboration remains paramount to optimize postoperative outcome in this complex pathology.

## 1 Introduction

Primary aortic sarcoma, classified within the broader family of soft tissue sarcomas (STS), is a rare and devastating malignancy with poor survival. While aortic sarcoma encompasses various histologic subtypes commonly seen in STS, its location within the aortic wall and need for complex resection presents unique challenges and makes these cases particularly lethal. Descriptions are largely confined to case reports and series, though pooled analysis suggests a median survival of 11 months with a significant portion of patients undergoing both surgical resection and concomitant vascular reconstruction [1]. Histopathologic reports have further identified that many arise from the intimal layer and may involve any portion of the aorta with the abdominal aorta and thoracic aorta as the most common locations [2]. Given the paucity of reports, we present a case series of patients with pathologically confirmed aortic sarcoma. Notably, we describe 3 distinct clinical scenarios: primary resection, resection after prior thoracic endovascular aortic repair (TEVAR), and nonoperative management after prior TEVAR.

## 2 Case 1

### 2.1 Patient Presentation

A 33‐year‐old man presented with dull abdominal pain. His history was significant for mediastinal Hodgkin's lymphoma treated with six cycles of ABVD chemotherapy and radiation 3 years prior. MRA identified a 3.2 × 2.8 cm partially circumferential mass of the descending thoracic aorta. PET‐CT demonstrated areas of mild uptake without evidence of metastatic disease. Endoscopic ultrasound‐guided biopsy was non‐revealing, but video‐assisted thoracoscopic biopsy identified synovial cell sarcoma. The patient underwent four cycles of neoadjuvant ifosfamide with greater than 50% reduction in mass before operative resection.

### 2.2 Operative Approach

An incision was made overlying the eighth intercostal interspace. The distal thoracic aorta was freely dissected and intercostal arteries preserved when able. The left‐sided crural fibers of the diaphragm were reflected, exposing the proximal abdominal aorta. Proximal control was obtained at the mid‐thoracic aorta and distal control below the celiac artery. The aorta and tumor were mobilized with the surrounding tissue and resected en bloc, with margins extending to the left mediastinal pleura, periosteum of the spine, and vena cava. An 18 mm polytetrafluoroethylene graft was sewn as an interposition bypass. The patient was discharged on postoperative day 16 after management of a chyle leak. Surgical pathology confirmed synovial sarcoma arising from the adventitia of the aorta without involvement of the attached diaphragm.

Surveillance CT at 6 months demonstrated local recurrence and metastases with confirmation after right pleural biopsy. The patient underwent one cycle of temsiroliums and cixutumumab with progression of disease, two cycles of gemcitabine and docetaxel with progression of disease, two cycles of temozolomide with progression of disease, and three cycles of trabectedin with progression of disease. The patient subsequently enrolled in hospice care and died 20 months after his index operation.

## 3 Case 2

### 3.1 Patient Presentation

A 48‐year‐old man presented with several months of worsening back pain. Five months prior, he had undergone a TEVAR for a possible intramural hematoma. PET‐CT identified an 8.9 × 4.5 cm hypermetabolic paraspinal mass at the level of T11‐T12. An additional 1.7 cm subcutaneous left flank mass was identified and ultimately confirmed as high‐grade sarcoma with smooth muscle differentiation after excisional biopsy.

### 3.2 Operative Approach

**Figure 1:**
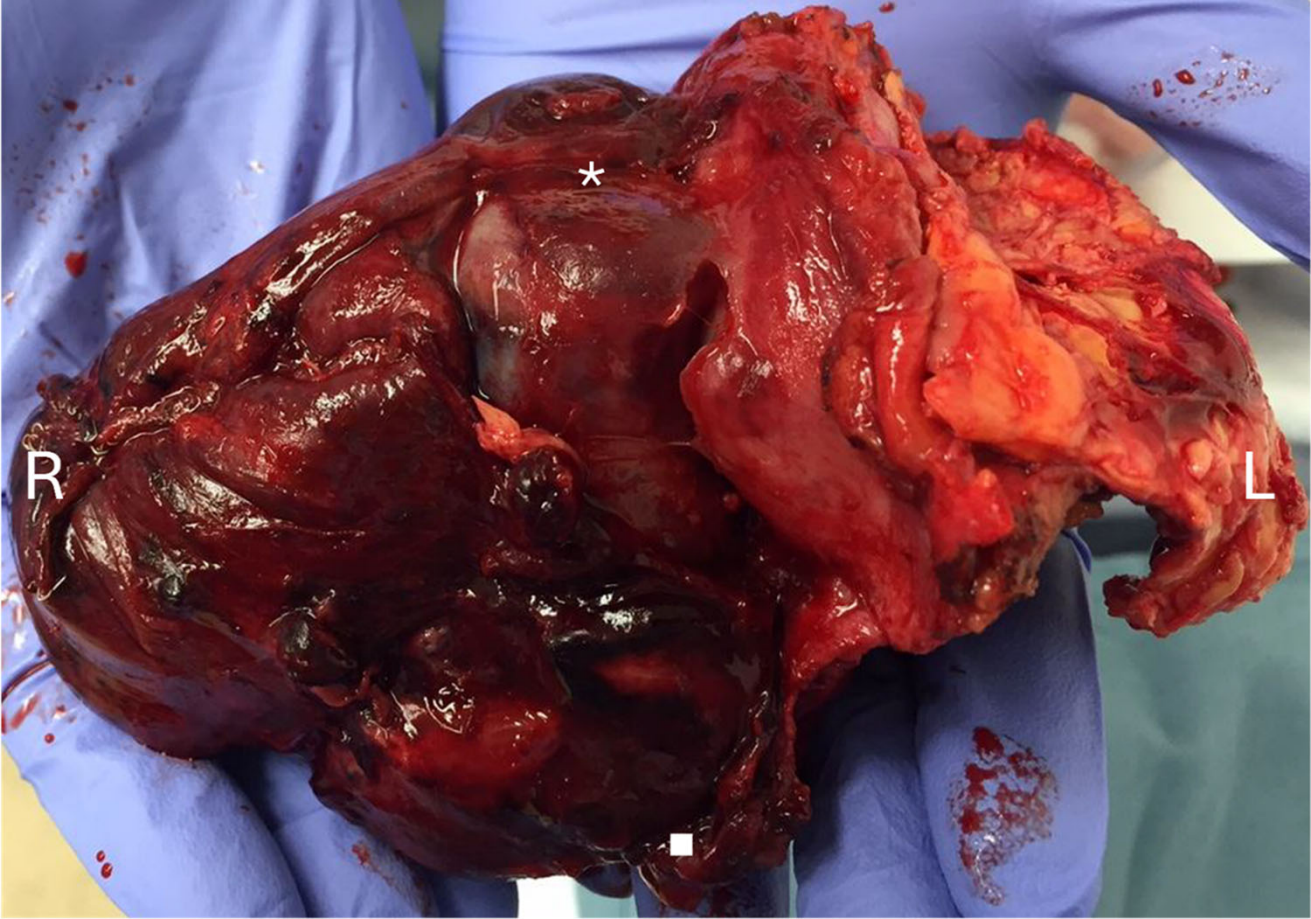
Intraoperative photo of explanted thoracic endograft and associated high‐grade aortic sarcoma. En bloc resection is shown with involved pleura visualized along right lateral border of endograft and emanating sarcoma along left lateral border. * = proximal stent graft. ▪ = distal stent graft.

An incision was made overlying the eighth intercostal interspace. Mobilization of the mass required a left lower lobectomy by thoracic surgery and vertebral body shaving by neurosurgery. After initiation of left heart bypass by cardiac surgery, the entire aorta and mass were resected en bloc (Figure 1). A 22 mm polyethylene terephthalate interposition graft was sewn from the thoracic aorta at the level of T8 to the supraceliac aorta. Postoperatively, the patient's course was complicated by acute on chronic left lower extremity ischemia necessitating thrombectomy, femoral endarterectomy, and femoral to above knee popliteal artery bypass on postoperative day 2. He was ultimately discharged on postoperative day 11.

Pathologic evaluation demonstrated nonspecific differentiation, though the relationship to the mediastinum and aorta was suggestive of an intimal sarcoma. The surgical margin was consistent with an R1 resection along the bone margin, and the patient underwent adjuvant radiation therapy. Five months postoperatively, he was noted to have micronodular metastases to bilateral lungs and a right corpus cavernosum mass consistent with metastatic sarcoma after biopsy. The patient established palliative chemoradiotherapy with his local oncologist and died 7 months after his index operation.

## 4 Case 3

### 4.1 Patient Presentation

**Figure 2:**
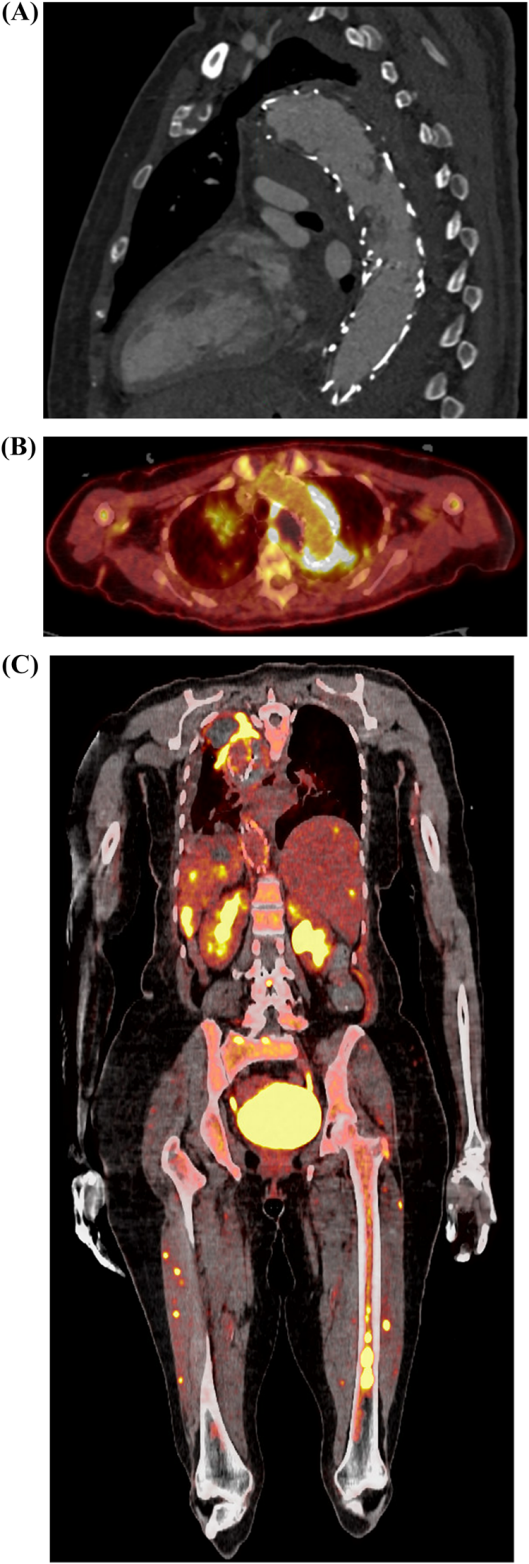
Cross‐sectional imaging of aortic sarcoma after prior thoracic endovascular aneurysm repair. (A) CTA sagittal view demonstrating luminal thrombus within endograft. (B) PET‐CT axial view demonstrating hypermetabolic activity around endograft. (C) PET‐CT coronal view demonstrating hypermetabolic activity of thoracic aorta, spleen, kidney, liver, and lower extremity osseous lesions.

A 64‐year‐old woman presented with epigastric pain and melena. Her history was significant for a TEVAR and left carotid‐subclavian bypass for a 5.8 cm thoracic aortic aneurysm 6 years prior. Her repair had been surveilled 9 months prior with stable sac diameter and improving bronchial‐based type 2 endoleak. CTA upon presentation identified new pedunculated thrombus within the endograft (Figure 2A), a focal 4.0 cm posterosuperior expansion of the aneurysm sac, and evidence of splenic, renal, and hepatic infarcts. The patient underwent an upper endoscopy with cauterization of gastric ulcers and eventually anticoagulated but with persistent transfusion requirements. On hospital day 16, the patient was taken for an exploratory laparotomy given concern for mesenteric ischemia on interval imaging. No ischemia was found but a jejunal polypoid mass was identified as the culprit of melena and subsequently resected. Pathology identified metastatic epithelioid angiosarcoma.

### 4.2 Hospital Course

Interval PET‐CT identified hypermetabolic nodular and soft tissue within the lumen and outside of the thoracic aortic endograft suggestive of tumor involvement (Figure 2B,C). Local invasion was identified in addition to metastases to liver, spleen, kidneys, and several osseous lesions. The patient was deemed a poor candidate for radiation therapy. The patient and family ultimately declined systemic therapy. The patient was transitioned to comfort care and died 6 weeks after presentation.

## 5 Discussion

Our case series illustrates the poor natural history of thoracic aortic sarcomas despite operative intervention. A distinct clinical feature of aortic sarcoma is the propensity for embolization, as seen in Cases 2 and 3 [1]. The use of TEVAR has been described to prevent such embolic events, though the deployment of endografts is limited to case reports and specific clinical scenarios [3]. Interestingly, Takamura et al. have previously described a postmortem diagnosis of angiosarcoma after TEVAR for thoracic aortic aneurysm [4]. Diagnosis was similarly challenging in their report with nonspecific sac expansion and type 2 endoleak. While Fatima et al. describe the explant of infrarenal endograft in the treatment of aortic sarcoma, to our knowledge Case 2 represents the first explant of a thoracic endograft for aortic sarcoma in the English literature [5]. Notably, in our case, en bloc resection of the mass necessitated simultaneous explantation of the endograft contained within the resected aorta. Nevertheless, as illustrated in this case series, long‐term survival remains dismal. In suitable candidates, we aim for complete resection with chemoradiotherapy for potential benefit on overall survival as described by other groups [5]. Specific to radiotherapy, our institutional practice typically foregoes neoadjuvant radiotherapy when complex resection and aortic reconstruction is required. Adjuvant radiotherapy is utilized in appropriate candidates with positive resection margin or local recurrence.

Given the complexity of these cases, a multidisciplinary tumor board discussion is essential for guiding management decisions, particularly in cases where the diagnosis is uncertain or in scenarios requiring explantation and complex reconstruction. A collaborative approach involving vascular surgery, cardiothoracic surgery, surgical oncology, medical oncology, radiation oncology, radiology, and pathology ensures comprehensive evaluation and facilitates individualized treatment planning. To further refine treatment strategies, larger retrospective studies analyzing outcomes across different treatment modalities, including surgical resection, endografting, and multimodal therapy, would be valuable. Such studies could help delineate prognostic factors and guide the development of standardized treatment algorithms. Additionally, further research is needed to evaluate any potential role for palliative endografting in patients who are not surgical candidates.

## Conflicts of Interest

M.K.E. is a paid consultant for W.L. Gore and Silkroad Medical. The remaining authors declare that the research was conducted in the absence of any commercial or financial relationships that could be construed as a potential conflict of interest.

## Synopsis

Case series review of primary aortic sarcomas.

## Data Availability

The data that support the findings of this study are available from the corresponding author upon reasonable request.
